# Preparation and evaluation of LA-PEG-SPION, a targeted MRI contrast agent for liver cancer

**DOI:** 10.1515/biol-2022-0074

**Published:** 2022-08-15

**Authors:** Lei Xia, Xiaowei Song, Guanghai Yan, Jishan Quan, Guangyu Jin

**Affiliations:** Department of Radiology, Affiliated Hospital of Yanbian University, No. 1327, Juzi Street, Yanji 133000, Jilin Province, P.R. China; Department of Anatomy, Basic Medical College, Yanbian University, Yanji 133000, Jilin Province, P.R. China; Department of Pharmaceutics, College of Pharmacy, Yanbian University, No. 977, Gongyuan Street, Yanji 133000, Jilin Province, P.R. China

**Keywords:** liver cancer, asialoglycoprotein receptor, superparamagnetic iron oxide nanoparticles, MRI contrast agent

## Abstract

This study aims to synthesize a magnetic resonance imaging (MRI) contrast agent that can specifically target the asialoglycoprotein receptor of liver cancer cells and evaluate its ability as a targeted MRI contrast agent. Lactobionic acid (LA) and polyethylene glycol (PEG) were used to modify superparamagnetic iron oxide nanoparticles (SPION) to obtain LA-PEG-SPION. LA-PEG-SPION was uniformly spherical under the electron microscope, with regular morphology and good dispersion. The particle size of LA-PEG-SPION was about 30 ± 4.5 nm, and its surface potential was about 31 ± 1.5 mV. LA-PEG-SPION had no toxicity or low toxicity to HepG2 cells and HeLa cells, even at 400 μg/mL. The uptake of LA-PEG-SPION by HepG2 cells was higher than that of SPION, with increased blue-stained particles. The fluorescent labeling rate of HepG2 cells reached 68.8%, which was higher than that of the control group. *In vitro,* MRI showed that the T2-weighted signal intensity of HepG2 cells was lower than that of the control group. Conclusively, LA-PEG-SPION nanoparticles are synthesized in a simple and efficient way. They are successfully applied to the T2-weighted contrast-enhanced MRI in liver cancer *in vitro*, and they have the potential to be used for *in vivo* research and clinical studies.

## 1 Introduction

Superparamagnetic iron oxide nanoparticle (SPION) is a new type of magnetic resonance imaging (MRI) contrast agent, which has good biodegradability and no accumulation in the body [1]. SPION can be used not only as an MRI contrast agent but also for magnetic thermotherapy and drug delivery systems through the effect of external magnetic fields [2]. In 1988, SPION was used for MRI of liver cancer because it could be taken up by Kupffer cells [3]. The high sensitivity and specificity of SPION as a targeted contrast agent can greatly improve the early diagnosis of liver cancer.

There are many surface modifiers of SPION. Polyethylene glycol (PEG) was first used to modify bovine serum proteins to change their immune properties [4], and is non-toxic and non-charged. It can avoid electrostatic interaction with plasma proteins and avoid recognition and engulfment by the reticuloendothelial system [5]. In addition, the molecular weight of PEG-modified SPION is significantly increased, exceeding the ability of glomerular filtration, and thereby, effectively increasing the circulation of SPION in the body [6]. Studies have shown that PEG-modified drugs have significant improvements in the following aspects: (1) increased solubility or dispersibility; (2) prolonged half-life; (3) reduced *in vivo* phagocytosis and enzymatic degradation, and (4) reduced toxicity [7,8]. SPION with different particle diameters has different effects. SPION, with a diameter of more than 200 nm, is easily taken up by the reticuloendothelial system of the liver and spleen and thus can be used as a contrast agent for the MRI of the liver and spleen, which is also called a passive targeted contrast agent [9]. SPION with a diameter of below 200 nm can avoid the phagocytosis of the reticuloendothelial system and achieve active targeting through binding with the surface receptors of diseased cells [10,11].

Asialoglycoprotein receptor (ASGP-R) is a receptor protein that is specifically expressed on the surface of liver cells. It can efficiently recognize ligands with galactose residues and promote their uptake by liver cells [12]. Studies have found a large amount of ASGP-R on liver cancer cells, and their expression levels are different among liver cancers of different malignant degrees [13,14]. ASGP-R is a receptor specifically expressed by normal and cancerous liver cells, and it is rarely expressed in other tissues [15]. Therefore, molecular probes mediated by ASGP-R can not only be used for targeted imaging of liver cancer but also for targeted imaging of some metastatic cancers originating from liver cancer or free liver cancer cells. Lactobionic acid (LA) contains galactose groups, which can bind to ASGP-Rs on the cell surface and serves as an exogenous specific ligand for active targeting of liver cancer [16]. It has the advantages of easy modification and low cost as a small molecule and is easy to couple with SPION. There has been no report on the preparation of a targeted MRI contrast agent for liver cancer using ASGP-R as the target.

In this study, a contrast agent, LA-PEG-SPION, for liver MRI was constructed, and its physical and chemical characteristics were evaluated. In addition, its effect on targeted imaging was examined by *in vitro* cellular experiments. Our findings provide experimental evidence for using LA-PEG-SPION in *in vivo* research and clinical practice.

## 2 Materials and methods

### 2.1 Synthesis and characterization of LA-PEG-SPION

First, the precursor LA-PEG-COOH was prepared. The carboxyl group on the surface of lactic acid was activated by 1-(3-dimethylaminopropyl)-3-ethylcarbodiimide hydrochloride (EDC)/*N*-hydroxysuccinimide (NHS) system, and then the lactic acid was combined with NH_2_-PEG-COOH by condensation reaction to form LA-PEG-COOH. SPION was fully dispersed in 20 mL of deoxygenated water and sonicated for 30 min. Then, 100 μL of 3-aminopropyltriethoxysilane (Sigma-Aldrich Co., St. Louis, MO, US) was slowly added dropwise under magnetic stirring. The whole process was protected by nitrogen, and the reaction was performed under stirring at room temperature for 24 h. An acylated SPION solid was obtained after dialysis and freeze-drying. LA-PEG-COOH and EDC were mixed and dissolved in 20 mL of ultrapure water, and the reaction was carried out under magnetic stirring at room temperature for 30 min. The above solution was slowly added dropwise to a beaker containing NHS, and the reaction was performed under stirring at room temperature for 3 h. The acylated SPION was fully dispersed in 20 mL of ultrapure water under ultrasonic vibration. Then, the two groups of solutions were evenly mixed, and the reaction was performed under magnetic stirring for 12 h with the protection of nitrogen. The reacted solution was dialyzed in a dialysis bag of MW8000 (Spectrum, CA, USA) at 4°C, and freeze-dried to obtain solid LA-PEG-SPION powder.

The morphology of LA-PEG-SPION was observed with an electron microscope at a working voltage of 80 kV (JSM-100S; Electronics Corporation, Japan). The particle size and surface potential of LA-PEG-SPION were measured by a laser particle size analyzer (Chengdu Jingxin Powder Testing Equipment Co., Ltd, Chengdu, China) and a precision potentiometric titrator (Metrohm Co., Switzerland), respectively.

### 2.2 MTT assay

HepG2 and HeLa cells were seeded to a 96-well plate (Corning, USA) at a concentration of 5 × 10^4^/mL and cultured in DMEM for 24 h. LA-PEG-SPION with different concentrations (25, 50, 100, 200, and 400 μg/mL) were fully dispersed in the serum-free culture medium and added to the wells. After 24 h of treatment, 20 μL of MTT solution (5 mg/mL; Aladdin, China) was added to each well and incubated under dark conditions for 4 h, according to the previous description [17]. Then, after washing, 150 μL of DMSO (Sigma, St. Louis USA) was added to each well. The optical density of each well at 490 nm was measured using a microplate reader (Tecan Sunrise, Switzerland). The untreated cells were used as a control. The absorbance of untreated cells was used as the reference value for calculating 100% cell viability. All experiments were repeated in triplicate with three parallel samples in each measurement.

### 2.3 Prussian blue staining

HepG2 cells were cultured on coverslips in 6-well plates with 5 × 10^4^ cells per well. Each well was added with 20 μg/mL of SPION and LA-PEG-SPION. After culturing overnight, cells were washed and fixed with 2 mL of 4% paraformaldehyde. After washing again, Prussian blue staining was performed using the Prussian Blue Staining Kit (Solarbio life science, Beijing, China) according to the kit instructions. After that, slides were mounted with neutral gum and observed under a microscope (BX51FC, OLYMPUS, Tokyo, Japan).

*Preparation of the sample for cytometry*: HepG2 and HeLa cells were seeded into two 6-well plates at 5 × 10^5^ cells per well. In the 6-well plates, 9 wells were seeded with HepG2 cells, and 3 wells were seeded with HeLa cells. Three wells of HepG2 cells were set as control, three wells of HepG2 cells were set as competition control, and 1.08 mg of LA was added. After being cultured for 24 h, the other three wells of HepG2 cells were added with LA-PEG-fluorescein isothiocyanate (FITC) at a concentration of 20 μg/mL. Meanwhile, 20 μg/mL LA-PEG-FITC was also added to the competition control well and the wells with HeLa cells, respectively. The cells were cultured in the dark for 4 h, then collected and analyzed by flow cytometry.

### 2.4 Flow cytometry

Flow cytometry was performed as previously described [18]. Fluorescein FITC coupled to LA-PEG was used to verify the targeting function of the probe, and flow cytometry (Thermofisher, USA) was used to detect the fluorescence intensity of cells in different groups. First, the blank group was analyzed to record the fluorescence distribution of blank control cells and draw the target area for comparison. Then, 0.36 mg/mL HepG2 cell group, 0.36 mg/mL HeLa group, 0.54 mg/mL HepG2 group, 0.54 mg/mL Hela group, and competition group were added sequentially. The fluorescence labeling rate of each group was recorded.

### 2.5 *In vitro* MRI

HepG2 and HeLa cells were seeded and cultured as described above. One well of HepG2 cells and one well of HeLa cells were used as control without contrast. The remaining two wells of HepG2 cells and HeLa cells were added with 20 and 40 μg/mL of LA-PEG-SPION suspension, respectively. After 4 h of incubation, NaOH solution (0.01 M) was added to lyse the cells. The cell fragment was mixed with a layer of 1% agarose gel, and a total of 500 μL of each sample was placed in a tube. After cell preparation, MRI was performed with a Siemens 3.0T MRI scanner for horizontal axis scanning. A T2 weighted sequence was performed. The specific parameters were: TR = 2,300, TE = 88, layer thickness = 0.6 mm, number of excitation = 1, field of view = 12 cm × 12 cm, matrix = 320 × 300. The region of interest of the same area was delineated in the same layer, and the T2WI signal intensity of three consecutive layers in each tube was measured [19].

## 3 Results

### 3.1 Synthesis and characterization of LA-PEG-SPION

**Figure 1:**
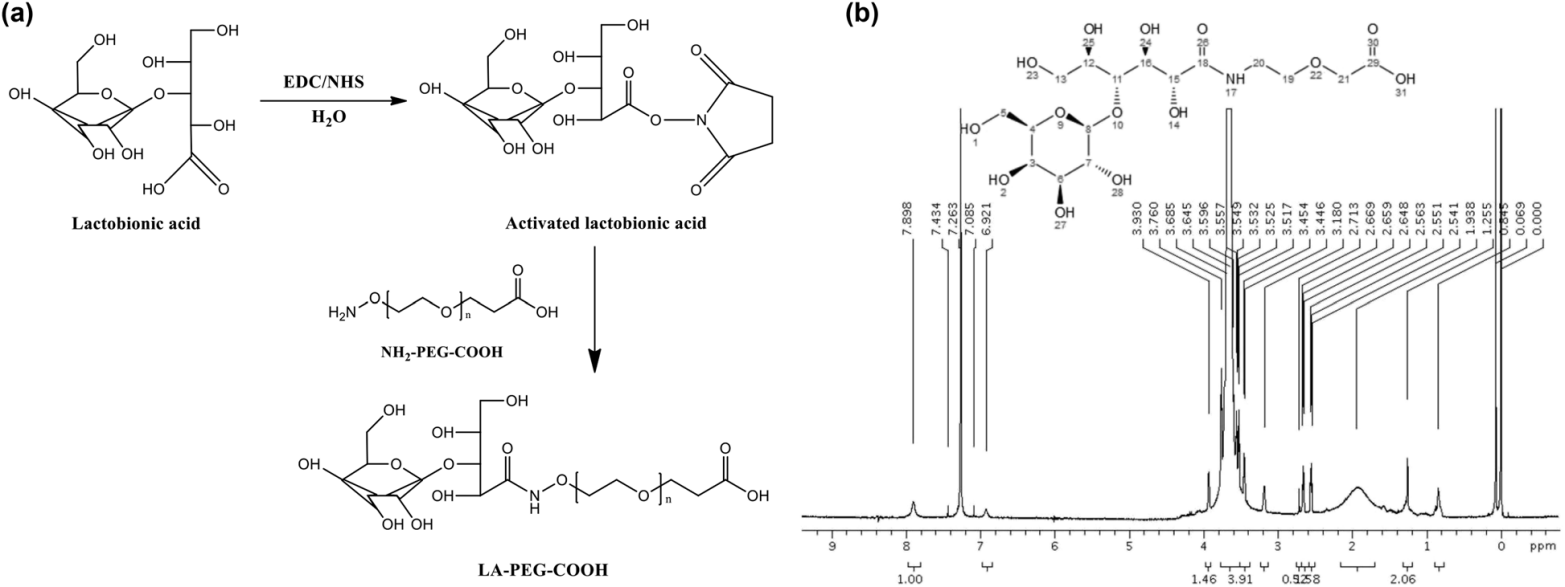
Reaction equation and ^1^H-NMR. (a) The reaction equation of LA-PEG-COOH and (b) the ^1^H-NMR of LA-PEG-COOH.

The reaction equation of LA-PEG-COOH is shown in Figure 1a. The ^1^H-NMR was used to verify the chemical structure of the synthesized LA-PEG-COOH (Figure 1b). The chemical bonds in the LA-PEG-COOH chemical structure were numbered 1–31. According to the analysis, the peak near 7–8 ppm in the map belonged to No. 17 NH bond, and the peak near 3–4 ppm represented the chemical bonds 1, 2, 27, 28, 25, 24, and 23, respectively. The peak near 2–3 ppm represented the chemical bonds 13, 6, 7, 8, 4, 5, 12, 11, 20, and 19, respectively. The result of ^1^H-NMR demonstrated that LA-PEG-COOH was successfully synthesized.

**Figure 2:**
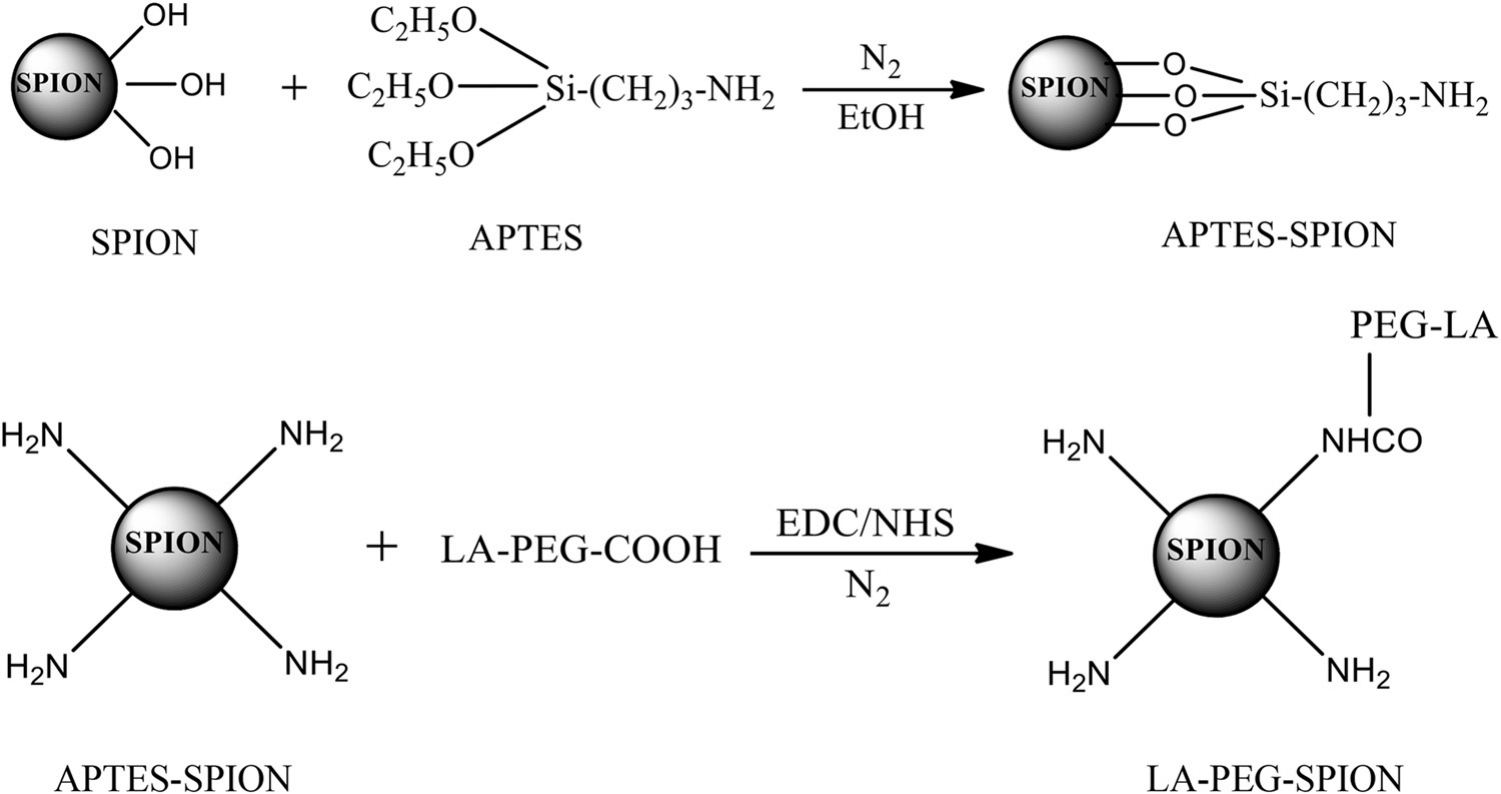
The reaction equations for LA-PEG-SPION synthesis.

LA-PEG-SPION was synthesized by the condensation reaction of LA-PEG-COOH and SPION using the chemical coupling agent EDC/NHS (Figure 2). Zeta potential analysis of SPION and LA-PEG-SPION showed that the surface potential of SPION was approximately (28 ± 1.5) mV, while that of LA-PEG-SPION increased slightly, to approximately (31 ± 1.5) mV. This suggests that both SPION and LA-PEG-SPION can form stable magnetic fluids in dispersed systems.

**Figure 3:**
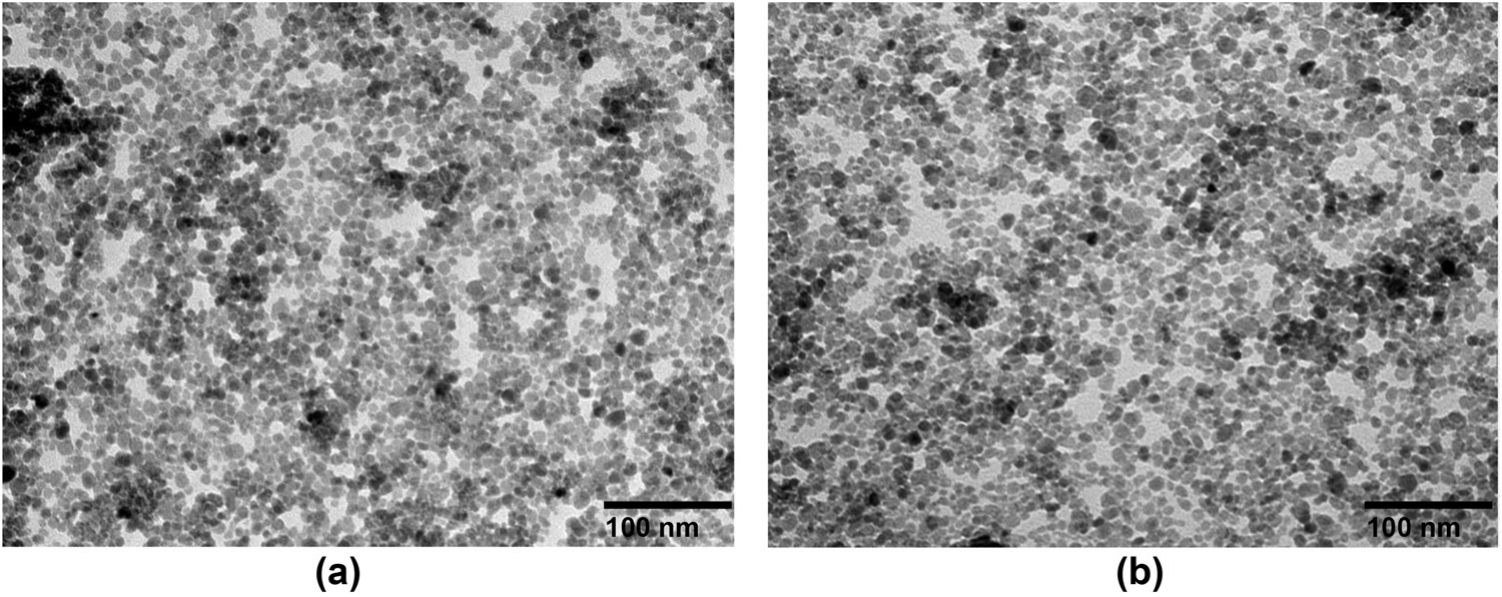
TEM images of (a) LA-PEG-SPION (magnification: 30k×) (scale bar = 100 nm) and (b) SPION (magnification: 30k×) (scale bar = 100 nm).

The electronic microscopic images showed that SPION and LA-PEG-SPION were both spherical particles. The LA-PEG-SPION particles were relatively uniform, with a core particle diameter of about 13 ± 1.5 nm (Figure 3a). No apparent agglomeration was observed. In addition, thin blurred aperture shadows were observed on the surface of the particles. This is because a thin film was formed on the particle surface after SPION was modified with LA-PEG. On the other hand, SPION was evenly dispersed in water without evident agglomeration (Figure 3b). The core particle size was about 10 nm, which was slightly smaller than LA-PEG-SPION. This may be related to the modified layer. These results indicate that the LA-PEG-SPION has a regular morphology, and its core particle size increases slightly, which is still in the appropriate range.

### 3.2 Cytotoxicity of the particles

**Figure 4:**
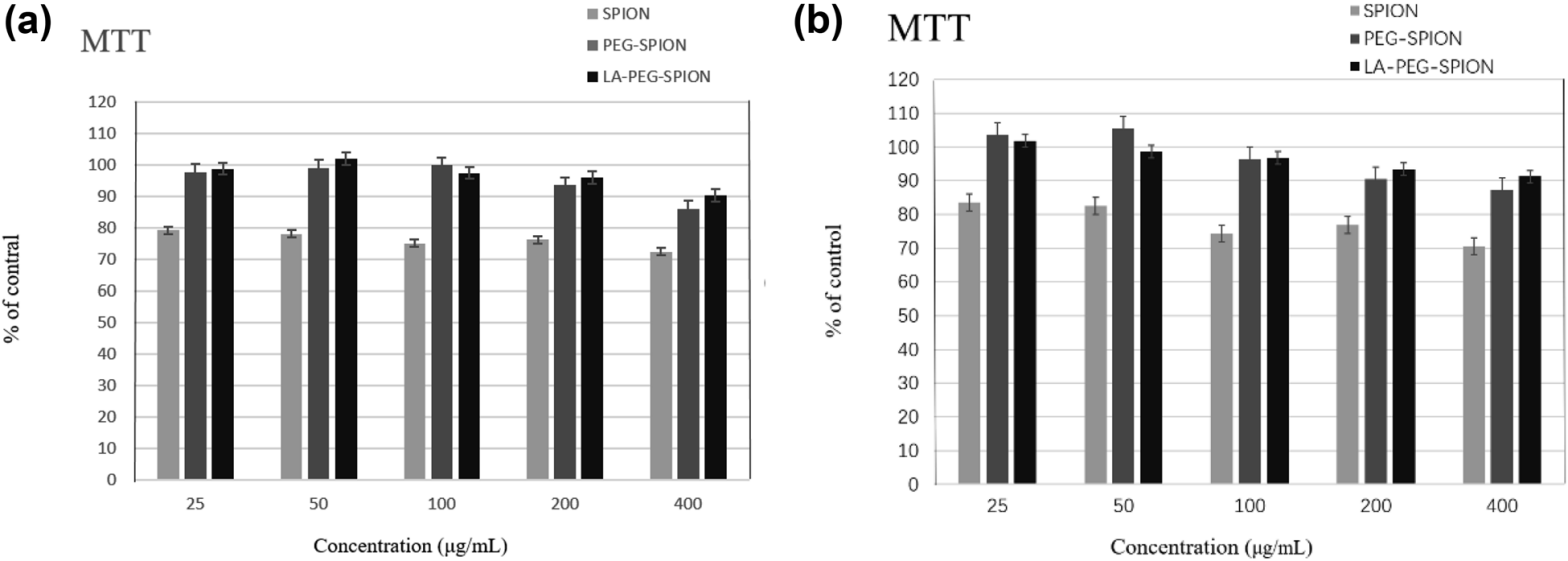
Viability of (a) HepG2 and (b) HeLa cells detected by the MTT assay. The cells were treated with different concentrations (25, 50, 100, 200, and 400 μg/mL) of LA-PEG-SPION for 24 h.

MTT method is more widely used in the toxicity analysis of molecular imaging probes than some new cytotoxicity experiments [20]. To determine the cytotoxicity of the particles, an MTT assay was performed. As shown in Figure 4, both HepG2 and HeLa cells had high viability after treatment with various concentrations (25–400 μg/mL) of SPION, PEG-SPION, and LA-PEG-SPION, suggesting no evident cytotoxicity on both HepG2 and HeLa cells. Cells treated with PEG-SPION and LA-PEG-SPION had higher viability than those with SPION, indicating that PEG-SPION and LA-PEG-SPION may have lower cytotoxicity than SPION. In addition, at a high concentration of 400 μg/mL, the viability of HepG2 and HeLa cells treated by LA-PEG-SPION was as high as 90%, which shows that SPION, PEG-SPION, and LA-PEG-SPION are non-toxic or low-toxic at high concentrations. These results indicate that the modification of SPION, especially by PEG with high biocompatibility, could significantly reduce the cytotoxicity of the nanoparticles.

### 3.3 The SPIONs are taken up by liver cells

**Figure 5:**
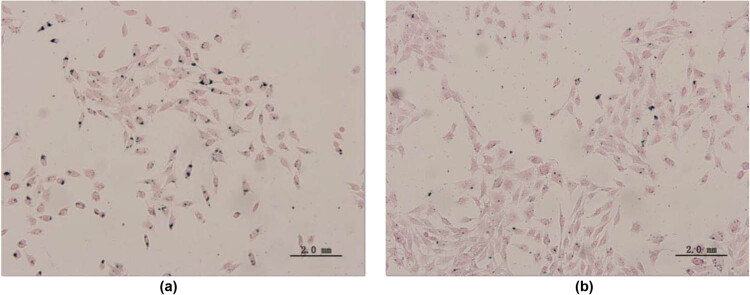
Images of Prussian blue staining. Prussian blue staining of (a) HepG2 and (b) HeLa cells cultured with LA-PEG-SPION.

To observe the uptake of SPION by cells, Prussian blue staining was performed. HepG2 cells were treated with 20 μg/mL LA-PEG-SPION or SPION. As shown in Figure 5, a large amount of blue-dyed iron particles were observed in the HepG2 cells treated with LA-PEG-SPION, while the cells treated with SPION had only a few blue particles. This indicates that LA-PEG-SPION is easier to be taken up by HepG2 cells than SPION, and that, to a certain extent, LA-PEG-SPION may have a targeting effect on liver cancer cells.

### 3.4 Cell uptake of FITC labeled particles

**Figure 6:**
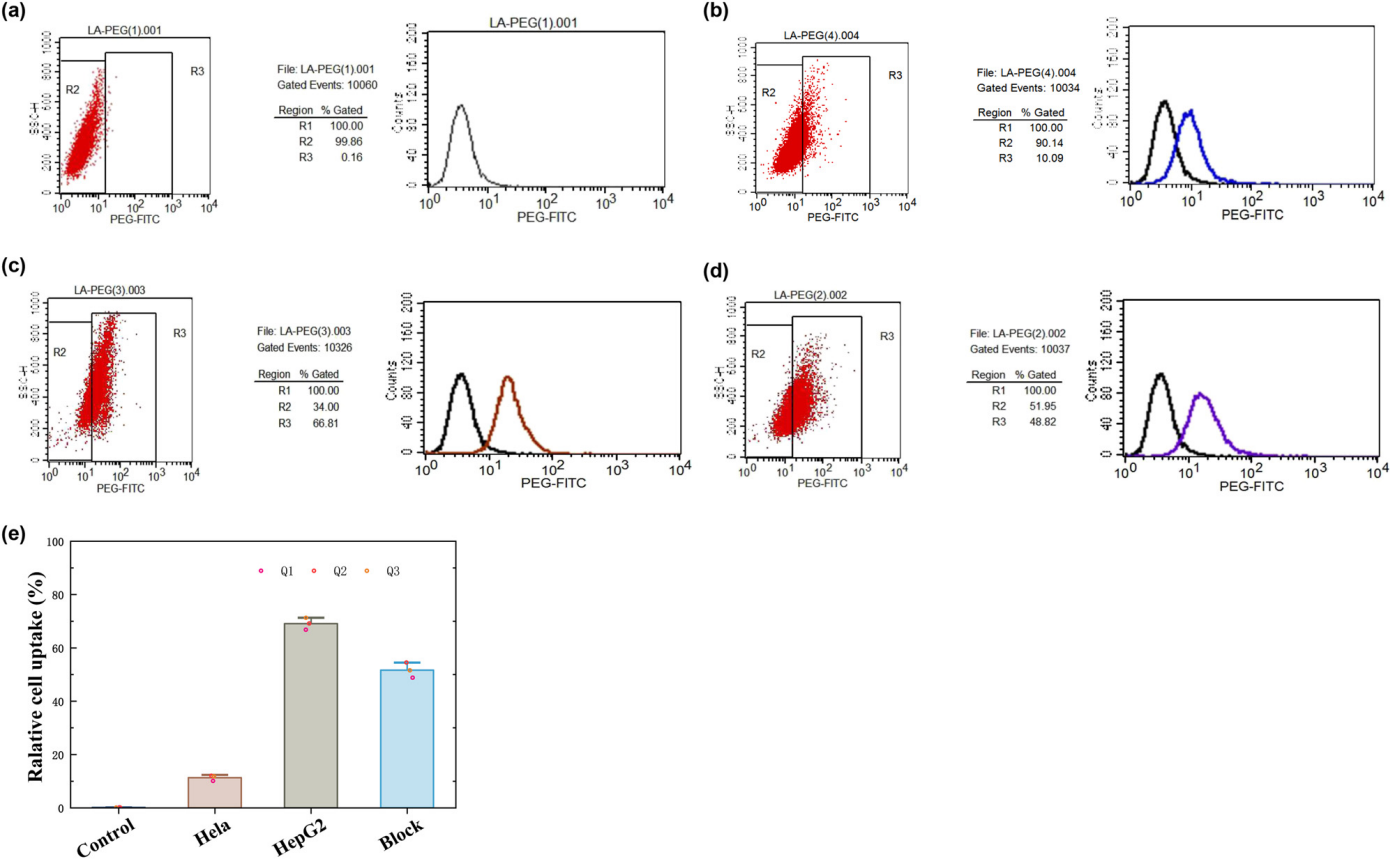
Flow cytometry for the uptake of fluorescent markers by cells. (a) Blank control; (b) HeLa cells; (c) HepG2 cells; and (d) LA competition group. (e) Quantification of the cell uptake of LA-PEG-SPION.

To detect the uptake of fluorescent labeled particles by cells, flow cytometry was performed. The results showed that no fluorescence was detected in the HepG2 cells of the blank control group (Figure 6a). The HeLa cells incubated with 20 μg/mL LA-PEG-FITC had an uptake rate of only 10.09% (Figure 6b and e), while that of the HepG2 cells was as high as 66.81% (Figure 6c and e). The HepG2 cells of the competition group showed a fluorescence labeling rate of 48.82% (Figure 6d and e), which was lower than that of the non-competitive group. This may be because the concentration of the drug used was not high enough to occupy all the ASGP-R receptors. The flow cytometry results showed that the order of the uptake rate of LA-PEG-FITC in the cells was HepG2 cells > HepG2 cells of the competition group > HeLa cells. This indicates that LA-PEG-FITC highly targets the ASGP-R high-expressing tumor cells.

### 3.5 LA-PEG-SPION shows targeted MRI imaging

**Figure 7:**
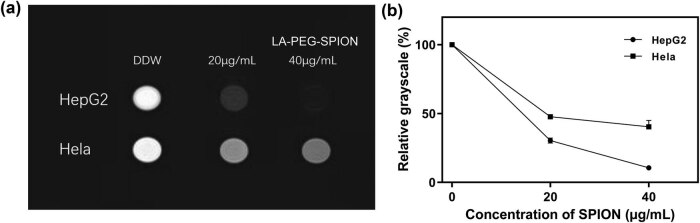
*In vitro* MRI. (a) T2WI images of *in vitro* MRI. (b) Quantification of the MRI signal by gray value. DDW, deuterium depleted water.

To verify the tumor-targeted ability of LA-PEG-SPION in MRI contrast imaging, the MRI enhancement test *in vitro* was performed. As shown in Figure 7, the signal intensities of the blank control groups of the two cell lines were basically the same, and their brightness was not evidently different from that of the ultrapure water control group. With the increase in LA-PEG-SPION concentration, the image brightness of HepG2 cells decreased. The relative gray value was lower than that in the control group. At a concentration of 40 μg/mL, the cell image brightness was extremely low, and the outline of the border could not be clearly observed on a black background. The brightness of HeLa cell images was not apparently decreased with the increase in LA-PEG-SPION concentration. As a comparison, the two cell lines showed apparent differences in image signal intensity at the same LA-PEG-SPION concentration. The HepG2 cells showed an evidently darker image than the HeLa cells, indicating that its T2-weighted signal intensity decreases more and that the uptake of LA-PEG-SPION by HepG2 cells is higher than HeLa cells.

## 4 Discussion

The average particle diameter of SPION was 30 ± 3.5 nm, while the particle diameter of LA-PEG-SPION was 33 ± 5.5 nm, which was slightly larger than that of SPION. This may be due to the fact that a thin coating layer was formed in solution after SPION was modified with LA-PEG, which slightly increases the hydrated particle size of the product. Studies have shown that SPION should have a nucleus particle size of about 10 nm, but the smallest pore size of permeable microvessels in normal tissues is about 10 nm. If the particle sizes are too small, there will be drug leakage, affecting their effects [21,22]. Therefore, special treatment is needed for SPION to obtain a suitable size for use. According to the electron microscope image, the core particle diameter of LA-PEG-SPION was about 10 nm, which meets the requirements of superparamagnetism. The hydrated particle size measured by the laser particle sizer is in line with the true particle size of the nanoparticles in the solution. Gupta and Gupta [23] showed that modified magnetic nanoparticles with a particle size between 10 and 100 nm had good stability and superparamagnetism, and thus were the most effective drug delivery carriers.

Lactic acid has a high affinity for ASGP-R, and PEG can effectively prolong the half-life of drugs *in vivo* and improve biocompatibility. Meanwhile, PEG can also reduce the formation of protein crowns and avoid the covering of active targeting groups. Flow cytometry results showed that the uptake of LA-PEG in hepatocellular carcinoma cells was higher than that in cervical cancer cells, and the cell labeling rate was more than 66%, even at a lower dose (20 μg/mL). The results of MR enhancement imaging of LA-PEG-SPION *in vitro* were consistent with that of flow cytometry. This part demonstrates that a new type of actively targeted magnetic resonance contrast agent has been constructed, which can provide a reference for the development of MR molecular imaging.

The safety of contrast agents is the most important prerequisite for its clinical application. In this study, the cytotoxicity of LA-PEG-SPION was detected by the MTT method. The results showed that LA-PEG-SPION had no evident toxicity to HepG2 and HeLa cells, and the toxicity of LA-PEG-SPION was lower than that of SPION, which might be due to the high biocompatibility and safety of PEG. PEG not only prolongs the retention time of the drug in blood but also protects human tissues [24,25]. In this study, Prussian blue staining and flow cytometry were used to detect the targeting ability of LA-PEG-SPION for liver cancer cells. Potassium ferricyanide in Prussian blue dye can combine with iron particles to form blue insoluble ferricyanide Prussian blue. This is also a major feature of SPION. It can visually display the distribution of probes from the tissue level, which is not available in other types of nanoparticles.

## 5 Conclusion

In this study, an MRI contrast agent LA-PEG-SPION that could specifically target liver cancer cell surface receptor ASGP-R was successfully synthesized. It had a regular circular shape and a stable surface potential and could form magnetic fluid in solution. It was low-toxic or non-toxic to HepG2 and HeLa cells. *In vitro* experiments showed that LA-PEG-SPION had specific targeting for liver cancer cells and may serve as a potential T2-weighted negative contrast in MRI. LA-PEG-SPION has the necessary conditions to become a targeted MRI contrast agent for liver cancer, and this study provides a basis for further *in vivo* evaluation and clinical studies.
